# Characterization of *Lactobacillus rhamnosus* MP01 and *Lactobacillus plantarum* MP02 and Assessment of Their Potential for the Prevention of Gastrointestinal Infections in an Experimental Canine Model

**DOI:** 10.3389/fmicb.2019.01117

**Published:** 2019-05-24

**Authors:** Leónides Fernández, Raquel Martínez, Manuela Pérez, Rebeca Arroyo, Juan M. Rodríguez

**Affiliations:** ^1^Departmental Section of Food Technology, Complutense University of Madrid, Madrid, Spain; ^2^Veterinary Center “Galileo”, Madrid, Spain; ^3^Department of Nutrition and Food Science, Complutense University of Madrid, Madrid, Spain

**Keywords:** *Lactobacillus rhamnosus*, *Lactobacillus plantarum*, dog, probiotics, gastroenteritis, prevention

## Abstract

The aim of the present study was to evaluate the probiotic potential of *Lactobacillus rhamnosus* MP01 and *Lactobacillus plantarum* MP02, two strains isolated from canine milk. These two isolates were characterized *in vitro* for their survival to conditions similar to those found in the canine gastrointestinal tract, production of antimicrobial compounds, adherence to intestinal mucin, degradation of mucin, and antibiotic sensitivity. Globally, both strains exhibited a high *in vitro* probiotic potential. Finally, their potential for the prevention of gastrointestinal infections was evaluated in an experimental canine model using 1-month-old puppies. A group of 12 German shepherd puppies, 6 males and 6 females, received *L. rhamnosus* MP01 daily for 2 months and a second group of 12 puppies, 6 males and 6 females, of the same breed received *L. plantarum* MP02 during the same period of time. The same experimental approach was carried with Yorkshire puppies. Additionally, the trial included 12 dogs of each breed in the placebo groups. The results demonstrated that administration of the strains resulted in a significant preventive effect of gastrointestinal infections in such populations.

## Introduction

Infectious gastroenteritis is one of the most common conditions in canine practice, being particularly frequent among puppies. A wide spectrum of bacteria and viruses are typically associated with diarrhea in puppies and young dogs (Magne, 2006; Schulz et al., 2008; Marks et al., 2011). Diarrheal episodes ranges from mild to severe cases and, while mild infections may recover without the use of antibiotics, empiric antibiotherapy is often prescribed in practice.

Antibiotics have contributed significantly to the improvement of animal health but their routine use in pets has raised two main concerns. The first concern is the potential development and spread of antibiotic-resistant bacteria that may compromise the treatment of animal and human infectious diseases in the future (Lloyd, 2007). Isolation of antibiotic-multiresistant strains as agents of nosocomial infections in dogs hospitalized in intensive care units is increasing (Guardabassi et al., 2004) and involves species as relevant as *Acinetobacter baumannii*, *Staphylococcus aureus*, *Escherichia coli*, or *Salmonella enterica*. The transmission of such organisms may occur between pets but, also, between pets, owners and veterinary personnel (Manian, 2003; Gandolfi-Decristophoris et al., 2013).

The second concern is the impact of wide spectrum antibiotics on the gut microbiota of the host, especially in relation to puppies since they may interfere with normal acquisition of the gut microbiota in early life, a process that has been linked to relevant short and long term health effects in humans. In the last few years, there has been an emerging interest in the role of the gastrointestinal (GI) microbiota in canine health and disease, from infectious or parasitic diarrheal diseases to inflammatory bowel diseases (Vázquez-Baeza et al., 2016; Redfern et al., 2017). The canine GI tract harbors a highly complex microbiota, which comprises not only bacteria but, also, fungi, viruses and protozoa. Although the normal composition and roles of the canine gut microbiome is far from elucidated, the loss of normal commensal bacteria in acute and chronic GI diseases has been linked to metabolic and immunological changes (Guard et al., 2015), highlighting the importance of dysbiosis in the pathophysiology of such diseases (Suchodolski, 2016).

In this context, strategies to reduce antibiotic use and/or mitigate their adverse effects on the host microbiota must be developed. In contrast to human gastroenterology, the use of probiotics in canine practice has been scarce. The objective of this work was the study of the potential of two lactobacilli strains isolated from canine milk to prevent gastroenteritis episodes in a canine experimental model.

## Materials and Methods

### Isolation and Identification of *Lactobacillus rhamnosus* MP01 and *Lactobacillus plantarum* MP02 From Canine Milk

Milk was collected as previously described (Martín et al., 2010) from a healthy 4-years-old German shepherd bitch, with a normal pregnancy and delivery, at day 29 after delivery. The sample was kept on ice until delivery to the laboratory and processed within the first 1 h after collection. Milk samples were diluted with peptone water and 100 μl of diluted sample was spread in triplicate on Man, Rogosa, and Sharpe (MRS, Oxoid, Basingstoke, United Kingdom) agar plates supplemented with L-cysteine (0.5 g/L) (MRS-Cys). Then, the plates were incubated anaerobically (85% nitrogen, 10% hydrogen, 5% carbon dioxide) in a anaerobic workstation (MINI-MACS, DW Scientific, Shipley, United Kingdom) at 37°C for 48 h.

All isolates showing different colony morphologies were selected from the agar plates and transferred to MRS broth tubes, which were incubated aerobically. These conditions aimed to exclude fastidious isolates that require specific incubation requirements and, therefore, would not be suitable for successful applications. Two isolates that showed the best growth (∼9 log_10_ cfu/mL after overnight incubation at 37°C in aerobiosis) were identified by 16S rRNA gene sequencing following the procedure described by Kullen et al. (2000). An ABI PRISM^®^ BigDye^TM^ Terminator Cycle Sequencing kit and the AmpliTaq DNA polymerase were used to prepare all sequencing reactions following the manufacturer’s instructions (Applied Biosystems, Foster City, CA, United States). Sequencing reactions were run on an ABI 377A automated sequencer (Applied Biosystems). The sequences obtained were compared to 16S rRNA gene sequences in the EMBL database using BLAST algorithm. The identity of the strain was determined based on a percent identity score of > 98%.

### Survival of *L. rhamnosus* MP01 and *L. plantarum* MP02 After Exposition to Conditions Similar to Those Found in the Canine GI Tract

The survival of the lactobacilli strains when they were delivered using a commercial pet food was tested in an *in vitro* model simulating passage through stomach and small intestine as described by Marteau et al. (1997) with the modifications included by Martín et al. (2005). Portions of a pet food (50 g) containing approximately 10^9^ cfu/mL of the lactobacilli were vehiculated in 5 mL of a sterile electrolyte solution containing 6.2 g/L of NaCl, 2.2 g/L of KCl, 0.22 g/L of CaCl_2_, and 1.2 g/L of NaHCO_3_ to simulate the *in vivo* dilution by saliva. Then, canine gastric juice (5 mL) was added and the mixture was shaken at 37°C. The pH curve in the stomach-resembling compartment was controlled as described for monogastric mammals (Conway et al., 1987). The initial pH 5.0 of the mixture was adjusted sequentially to pH 4.1 after 20 min, pH 3.0 after 40 min, pH 2.1 after 60 min, and, finally, pH 1.8 after 80 min. Samples were successively removed at 0, 20, 40, 60, and 80 min, simulating the normal monogastric gastric emptying times (Marteau et al., 1997). After adjusting their pH to 6.5 ± 0.2 with 1 M NaHCO_3_, samples were mixed with 10 mL of simulated duodenal juice [a sterile electrolyte solution containing 5 g/L of NaCl, 0.6 g/L of KCl, 0.3 g/L of CaCl_2_, 4% of canine bile, and 7% of pancreatin (Sigma, St. Louis, MO, United States)] (Marteau et al., 1997). After 120 min of exposition, bacterial survival was determined by plating the samples onto MRS agar plates, which were anaerobically incubated at 37°C for 48 h. Canine gastric juice (chloride: 129 mmol/L; sodium: 68 mmol/L; pH: 3.4) and bile was provided by the Veterinary Faculty (Complutense University of Madrid, Spain).

### Determination of the Antimicrobial Activity of *L. rhamnosus* MP01 and *L. plantarum* MP02

An overlay method (Magnusson and Schnürer, 2001) was used to determine the ability of *L. rhamnosus* MP01 and *L. plantarum* MP02 to inhibit the growth of various species of bacteria. The lactobacilli were inoculated (approximately 2-cm-long lines) on MRS agar plates and incubated at 32°C for 48 h in anaerobic jars (Oxoid). Then, the indicator microorganisms (approximately 10^4^ cfu) vehiculated in 10 mL of soft (0.7% agar) BHI (Oxoid) were inoculated on top. The bacteria employed as indicator organisms (our own culture collection) were originally isolated from feces of dogs with gastroenteritis and included *Clostridium perfringens* MP24, *Enterococcus faecalis* MP33, *Staphylococcus aureus* MP29, *Escherichia coli* MP07 (O157:H7), MP11 and MP17, *Salmonella enterica* MP46, *Campylobacter jejuni* MP42, *Proteus vulgaris* MP48, and *Klebsiella pneumoniae* MP68. The plates overlaid with bacterial indicators were further incubated according to the optimal growth temperature of the indicator microorganism (32 or 37°C) for 48 h. Finally, the clear zones of inhibition (>2 mm) around the strain streaks were measured. All experiments assaying inhibitory activity were performed in triplicate.

Enzymatic methods were used to measure L- and D-lactic acid and acetic acid produced by both strains in MRS cultures. Specifically, the samples were assayed using commercially available enzymatic kits (Roche Diagnostics, Mannheim, Germany) according to the manufacturer’s instructions in triplicate. The pH values of the supernatants were also measured. Additionally, the antimicrobial activity of lactobacilli against the indicator bacteria was also assayed after the culture supernatants were neutralized to pH 6.2 with 1 M NaOH using an agar diffusion method described by Dodd et al. (1992).

The ability of both strains to produce bacteriocins was determined by an agar well diffusion assay as described by Martín et al. (2006). *Clostridium perfringens* MP24, *Enterococcus faecalis* MP33, *Staphylococcus aureus* MP29 were employed as indicators of bacteriocinogenic activity.

### Adherence to Epithelial Cells and Mucin

The adherence of lactobacilli to HT-29 and Caco-2 cells was examined basically as described by Coconnier et al. (1992). Routinely, cells were grown in DMEM medium (PAA, Linz, Austria) containing 25 mM glucose, 1 mM sodium pyruvate and supplemented with 10% heat-inactivated (30 min, 56°C) fetal calf serum, 2 mM L-glutamine, 1% non-essential amino acid preparation, 100 U/mL penicillin and 100 mg/mL streptomycin. For the adherence assays, HT-29 and Caco-2 were cultured to confluence in 2 mL of medium devoid of antibiotics. Approximately 10 days postconfluence, 1 mL of the medium was replaced with 1 mL of *Lactobacillus* suspension (10^8^ cfu/mL in DMEM). The inoculated cultures were incubated for 1 h at 37°C in 5% CO_2_. Then, the monolayer was washed five times with sterile PBS, fixed with methanol, stained with Gram stain and examined microscopically. The adherent lactobacilli in 20 random microscopic fields were counted for each test.

The adhesion of bacterial cells of both strains to mucin was determined according to the method described by Cohen and Laux (1995) and the modifications of Olivares et al. (2006). The assays were performed in triplicate and the values were expressed as the mean ± SD.

### Degradation of Gastric Mucin

The potential of the two lactobacilli strains to degrade partially purified hog gastric mucin (HGM; Sigma) *in vitro* was evaluated following the plate procedure developed by Zhou et al. (2001). A discolored halo around the colony after staining with 0.1% amido black in 3.5 M acetic acid for 30 min reveals mucin lysis. A fecal isolate was used as positive control culture. These assays were performed in triplicate.

### Sensitivity to Antibiotics

To analyze the susceptibility of the lactobacilli strains to different antibiotics, minimum inhibitory concentrations (MICs) to 18 antimicrobial agents were calculated using the Sensititre Staenc1F kit (Trek Diagnostic Systems Ltd, East Grinstead, United Kingdom) according to the instructions of the manufacturer. The following antimicrobial agents were tested: amoxicillin/clavulanic acid (AUG), ampicillin (AMP), chloramphenicol (CHL), ciprofloxacin (CIP), clindamycin (CLI), erythromycin (ERY), fosfomycin (FOS), gentamicin (GEN), imipenem (IMI), linezolid (LNZ), mupirocin (MUP), oxacillin (OXA), penicillin (PEN), quinupristin/dalfopristin (Q/D), rifampicin (RIF), teicoplanin (TEI), tetracycline (TET), trimethoprim/sulfamethoxazole (SXT). In addition, sensitivity to kanamycin (KAN), streptomycin (STR), and vancomycin (VAN) was tested using *E*-test strips (Biomerieux) and following the instructions of the manufacturer. The criteria followed to determine the sensitivity or resistance of the strains were those recently provided by EFSA (European Food Safety Authority [EFSA], 2018).

### Protective Effect Against Gastroenteritis in a Canine Experimental Model

A total of 72 animals (weaned 1-month-old puppies) belonging to two different breeds (German shepherd, *n* = 36; 18 males and 18 females; Yorkshire, *n* = 36; 18 males and 18 females) were recruited in the establishment of a dog breeder (El Molar, Madrid, Spain), which includes an in-house Veterinary Department and follows strict guidelines regarding animal health and welfare. Informed consent was obtained from the dog breeder. All the animals stayed in the establishment during the complete assay period since the dog breeder does not sell puppies until they reach an age of ≥ 4 months after birth. The animals were vaccinated at 6 weeks (distemper and parvovirus), 8 weeks (distemper, parvovirus, parainfluenza, hepatitis, leptospirosis), and 12 weeks (distemper, parvovirus, parainfluenza, hepatitis, leptospirosis) of age. All animals were treated in strict accordance with the guidelines of the European Directive 2010/63/UE on the protection of animals used for scientific purposes. The study was approved by Ethical Committee on Animal Experimentation of Universidad Complutense de Madrid (Spain), under protocol 29/17.

Puppies taken from different litters were assigned to either a diet supplemented with *L. rhamnosus* MP01 (LR group) or *L. plantarum* MP02 (LP group), or the same diet without any supplementation (control group). The assay was carried simultaneously for the three groups during late winter/spring. The diet was German Sherpherd Junior and Yorkshire Terrier Junior (Royal Canin) for the German shepherds and Yorkshire puppies, respectively. Probiotic supplementation was designed in order that all the animals of the LR and LP groups received ∼9 log_10_ cfu of *L. rhamnosus* MP01 or *L. plantarum* MP02, respectively, daily. Before starting the study, all the animals were submitted to a veterinary examination to exclude those with ongoing health disorders. Both the German shepherd and the Yorkshire puppies were distributed into one of the two study groups according to a randomization list generated by an informatic program, which had in account that the weight of German shepherd puppies, on one side, and that of the Yorkshire ones, on the other side, were similar at the beginning of the study. The incidence of gastroenteral infections for the 2-months duration of the study was recorded. The diagnosis of infectious diseases was made by a veterinary practitioner based on specific symptoms (e.g., presence of diarrhea). A GI infection was defined as loose or watery stools with or without fever or vomiting. Animals were weighed and their fecal samples were collected both at the beginning and at the end of the study.

Fecal samples were homogenized individually in a peptone-saline solution (100 mg/mL). To estimate the concentration of lactobacilli and enterobacteria, appropriate dilutions were spread in triplicate onto MRS-Cys agar plates (for lactobacilli), and MacConkey (MCK; Biomerieux) agar plates (for enterobacteria). MRS-Cys plates were incubated in anaerobiosis and MCK agar plates in aerobiosis; all the plates were incubated at 37°C for 48 h. Quantitative real-time PCR (qPCR) assays were performed for *Faecalibacterium* spp., since previous studies have found that this genus is considered as a marker of good canine gut health (Herstad et al., 2018). Initially, a fraction of the fecal samples was centrifuged at 13,000 rpm for 20 min. Then, total DNA was isolated from the pellets using the QIAamp DNA Stool Mini Kit (QIAgen, Hilden, Germany) following a protocol described previously (Martín et al., 2007). DNA was eluted in 20 μl and the purified DNA extracts were stored at −20°C.

The PCR assays (primers and conditions) were performed and the results were expressed as described previously (Garcia-Mazcorro et al., 2012). The DNA concentration of all fecal samples was adjusted to 5 ng μL^−1^. A commercial real-time PCR thermocycler (CFX96^TM^, Bio-Rad Laboratories, Hercules, CA, United States) was used for all experiments. Standard curves using 1:10 dilutions of DNA (ranging from 2 ng to 0.2 pg) from *Faecalibacterium prausnitzii* ATCC 27766 were used to calculate the unknown bacterial genomic targets. Thresold cycle (Ct) values between 16.72 and 20.87 were obtained for this range of *F. prausnitzii* DNA (*R*^2^ = 0.9907). The Ct values measured for DNA extracted from non-target species (*Bifidobacterium bifidum* ATCC 11863; *L. rhamnosus* MP01 and *L. plantarum* MP02) were 39.82 ± 0.59. All samples and standards were run in triplicate.

Fecal samples were assayed for IgA by ELISA (Bethyl Laboratories, Montgomery, AL, United States), as previously described (Vilson et al., 2018). Analysis of short-chain fatty acids (SCFAs; i.e., acetate, propionate, and butyrate) was performed using a dilution gas chromatography-mass spectrometry (GC-MS) assay as previously described (Moreau et al., 2003; Guard et al., 2015).

### Statistical Analysis

Normally distributed data are reported by means and 95% confidence intervals (CI) and non-normal distributed data by medians and interquartile ranges (IQR). Exploratory multifactorial or two-way ANOVA tests were performed to assess globally the impact of the supplementation with probiotics, breed and/or sex. One-way ANOVA tests were used to compare the mean values of the different variables between the three groups of dogs. Tukey’s HSD *post hoc* tests were performed when required to identify which specific group’s means were different after comparing all pairs of means. Paired *t*-tests allowed to compare the changes at individual level from the beginning to the end of the assay in the canine experimental model. The difference in the change of GI infection episodes between the control and the two probiotic groups (LP and LR) was tested using Fisher’s Exact Test for r × c tables (specifically, a 4 × 3 table)^1^.

The association between fecal IgA, acetate, propionate and butyrate and *Lactobacillus*, *Enterobacteriaceae* and *Faecalibacterium* concentration in feces was assessed using the Spearman’s rank correlation coefficient (two tailed).

Analyses were conducted using Statgraphics Centurion XVIII version 18.1.06 (Statgraphics Technologies, Inc., The Plains, VA, United States).

## Results

### Isolation and Identification of Lactobacilli From the Canine Milk Sample

Identification by 16S rRNA gene sequencing of the two isolates that showed the best growth revealed that they belonged to the species *L. rhamnosus* and *L. plantarum*, respectively. Since both species are included in the QPS list (European Food Safety Authority [EFSA], 2017), they were submitted to a further characterization.

### Survival of *L. rhamnosus* MP01 and *L. plantarum* MP02 After Exposition to Conditions Similar to Those Found in the Canine GI Tract

*Lactobacillus rhamnosus* MP01 and *Lactobacillus plantarum* MP02 showed a significant survival rate (∼45%) under simulated canine GI conditions. In the first step, bacteria were exposed to a secretion resembling saliva. This artificial fluid did contain neither lysozyme nor amylase because previous assays showed that they did not adversely affect their viability when added at the physiological levels found in the salivary secretion. The survival of both strains after simulated gastric digestion in the different fractions that were taken from the gastric-like compartment at 0, 20, 40, 60, and 80 min is shown in Table 1. Intestinal-like secretion had no effect on the population of both lactobacilli strains.

**Table 1:** Percentage (%) of the lactobacilli inoculated (10^9^ cfu) that survived to conditions simulating those of the canine gastrointestinal tract.

	Gastric-emptying fraction^a^				
Strain	20 min	40 min	60 min	80 min	% Total
*L. rhamnosus* MP01	15.43 ± 2.27	24.38 ± 2.56	18.09 ± 2.31	6.07 ± 0.60	63.97
*L. plantarum* MP02	15.96 ± 2.88	23.57 ± 2.99	15.02 ± 2.11	7.92 ± 0.73	62.47

*^a^Mean ± SD. The different fractions were taken from the gastric-like compartment at 0, 20, 40, 60, and 80 min and later submitted to the intestinal-like secretion.*

### Antimicrobial Activity

The mean inhibition zone around *L. rhamnosus* MP01 and *L. plantarum* MP02 streaks were > 2 mm indicating a noticeable inhibitory antimicrobial activity against all indicator organisms used in this study. This antibacterial effect was particularly effective against the Gram-negative strains. However, neutralization of the pH of the cultures of both strains led to the loss of the antimicrobial activity. The lactobacilli strains were also screened for production of bacteriocins but bacteriocinogenic activities could not be detected against the indicator strains under the assayed conditions.

### Other Properties

In this study, all the lactobacilli strains tested were strongly adhesive to both Caco-2 and HT-29 cells. The mean ± SD number of adherent *L. rhamnosus* MP01 cells in 20 random microscopic fields was 374.2 ± 102.5 and 907.2 ± 252.9 in Caco-2 and HT29 cells, respectively, while that of adherent *L. plantarum* MP02 cells was 334.6 ± 98.7 and 869.7 ± 241.1 in Caco-2 and HT29 cells, respectively. Both strains showed a strong adherence to mucin (approximately 12.5% of the fluorescence was retained in the wells after the washing steps of the assay), and were unable to degrade gastric mucin *in vitro*. The determination of antibiotics’ MICs revealed that both strains satisfy the EFSA criteria (European Food Safety Authority [EFSA], 2018) for antibiotic sensitivity of potentially probiotic bacteria (Table 2).

**Table 2:** Minimum inhibitory concentrations (MICs) and cut-off values (μg/ml) of a variety of antibiotics against *L. rhamnosus* MP01 and *L. plantarum* MP02.

		MICs	
Antibiotics	Cut-off values^∗^	*L. rhamnosus* MP01	*L. plantarum* MP02
Ampicillin	4	2	1
Clindamycin	4	0.5	2
Chloramphenicol	4	2	2
Erythromycin	1	0.25	0.5
Streptomycin	64	16	32
Gentamicin	16	1	1
Kanamycin	64	32	32
Tetracyclin	8	4	4
Vancomycin	n.r.	>64	>64
Linezolid	n.r.	1	0.5
Penicillin	n.r.	0.5	0.5

*^∗^European Food Safety Authority [EFSA] (2018); n.r., not required.*

### Effect of Probiotic Supplementation in the Canine Experimental Model

In this study 36 German Shepherd and 36 Yorkshire puppies were assigned to three groups: one control group (C group) that did not receive any intervention, and two probiotic groups that received either *L. plantarum* MP02 (LP group) or *L. rhamnosus* MP01 (LR group) at a dose of 10^9^ cfu/day for a 8-week period, starting at weaning (5 weeks of age). No differences were noted on the weight of the dogs (within each breed and sex) when the assay started (Table 3). Probiotic supplementation (experimental groups C, LP, or LR) and sex had a statistically significant effect on the weight gained by the German shepherd puppies at the end of the assay (two-way ANOVA; Supplementary Table S1). In contrast, sex was the only factor that had a significant effect on weight gain in Yorkshire puppies.

**Table 3:** Weight (kg) of German shepherd and Yorkshire puppies supplemented or not with the probiotics *L plantarum* MP02 or *L. rhamnosus* MP01 at the beginning (5 weeks of age) and at the end (12 weeks of age) of the assay in a canine experimental model.

				5 weeks of age		12 weeks of age	
Breed	Sex	Group^a^	*n*	Mean (95% CI)	Range (min-max)	Mean (95% CI)	Range (min-max)
German shepherd	Female	C	6	2.97 (2.75–3.18)	2.60–3.20	9.97 (8.82–11.12)	8.40–10.90
		LP	6	2.98 (2.84–3.12)	2.80–3.20	10.38 (9.80–10.97)	9.70–11.00
		LR	6	3.02 (2.81–3.22)	2.70–3.20	10.82 (9.95–11.68)	9.30–11.50
		*p*-value^b^		0.889		0.258	
	Male	C	6	3.47 (3.24–3.69)	3.20–3.80	11.58 (10.41–12.75)	10.70–13.20
		LP	6	3.47 (3.34–3.59)	3.30–3.60	12.05 (11.52–12.58)	11.40–12.70
		LR	6	3.43 (3.25–3.62)	3.20–3.70	12.67 (11.67–13.66)	11.20–13.60
		*p*-value		0.930		0.144	
Yorkshire	Female	C	6	0.34 (0.32–0.37)	0.31–0.37	0.68 (0.62–0.73)	0.61–0.74
		LP	6	0.36 (0.33–0.38)	0.33–0.39	0.71 (0.64–0.78)	0.63–0.80
		LR	6	0.35 (0.32–0.37)	0.32–0.38	0.71 (0.64–0.78)	0.63–0.80
		*p*-value		0.590		0.546	
	Male	C	6	0.49 (0.44–0.54)	0.41–0.56	0.93 (0.84–1.02)	0.78–1.02
		LP	6	0.48 (0.43–0.53)	0.43–0.54	0.94 (0.89–0.99)	0.87–1.02
		LR	6	0.48 (0.43–0.53)	0.42–0.55	0.95 (0.88–1.02)	0.85–1.04
		*p*-value		0.933		0.845	

*CI, confidence interval. ^a^C, Control group; LP, group supplemented with L. plantarum MP02; LR, group supplemented with L. rhamnosus MP01. ^b^One-way ANOVA tests to compare the mean weight of the puppies at each time point.*

**FIGURE 1:**
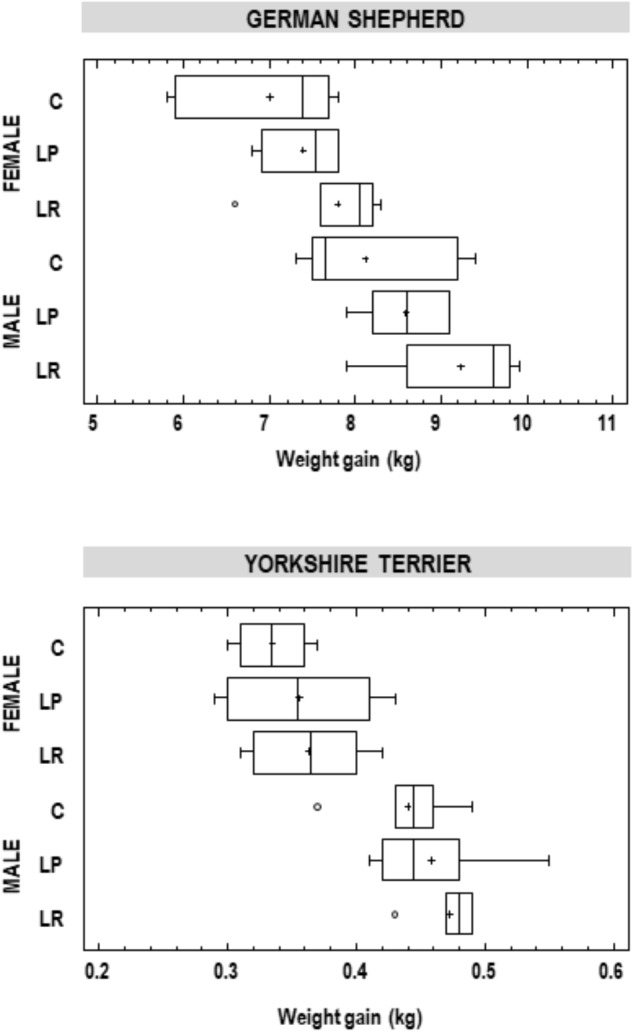
Weight gain (kg) in participant German shepherd and Yorkshire puppies at the end of the assay. C, control group; LP, group supplemented with *L. plantarum* MP02; LR, group supplemented with *L. rhamnosus* MP01.

When the body weight increase in animals was analyzed within each breed and sex, there was a high variation between individuals (Figure 1). Globally, the mean weight gained was always superior for male than for female puppies, and for those animals that had been supplemented with either of the two probiotics, but the differences were not statistically significant (one-way ANOVA; Figure 1). For German shephard puppies, the mean (95% CI) increase in body weight for the females in the control group was 7.00 (6.57–7.42) kg, while for the LP and LR groups of female puppies it was 7.40 (6.97–7.83) kg and 7.80 (7.37–8.23) kg, respectively. Similar results were registered for the other groups of puppies of different sex and breed (Figure 1). A trend in higher weight gain was identified in the group supplemented with *L. rhamnosus* MP01 when compared to the group supplemented with *L. plantarum* MP02 for both breeds and sexes, but these differences were not statistically significant (Figure 1).

**FIGURE 2:**
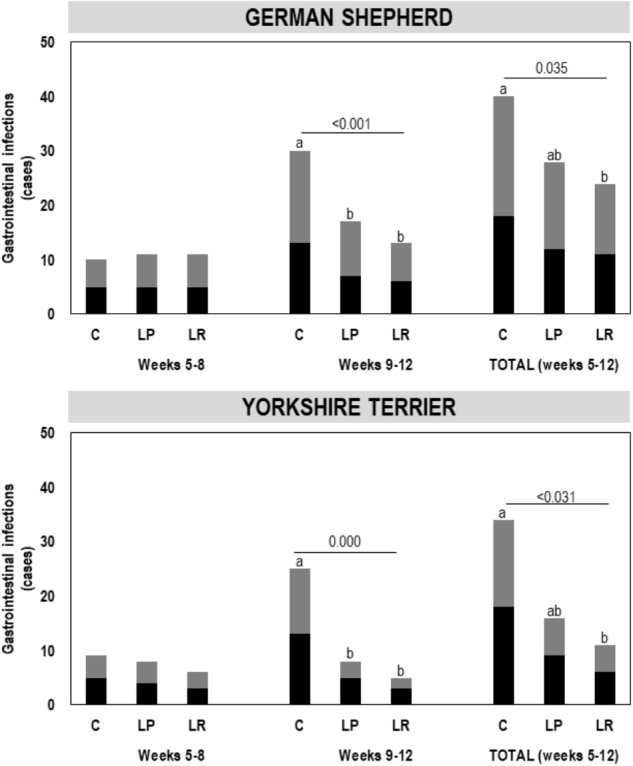
Gastrointestinal infection (GI) episodes registered for German shepherd and Yorkshire puppies during the first 4 weeks (weeks 5–8) and the last 4 weeks (weeks 9–12) of the assay, and the total number of episodes (weeks 5–12), in the control (C), *L. plantarum* MP02 (LP) and *L. rhamnosus* MP01 (LR) groups. There were statistically significant differences in the number of GI episodes registered during the last 4 weeks of the assay among the three groups (C, LP, and LR) for both breeds (one-way ANOVA). The letters above the bars indicate the statistically significant differences among the groups (Tukey’s HSD tests).

A total of 153 episodes of GI infections were registered during the assay (Figure 2). The mean (95% CI) number of GI infections among the puppies during the first 4 weeks (weeks 5–8 of age) of the assay was 0.76 (0.62–0.91) [total number of infections was 55 in this period], and no differences were found between the three groups (C, LP, and LR) within each breed (Figure 2). The total number of infections during the last 4 weeks of the assay raised to 98, and the mean (95% CI) number of GI infections per individual increased to 1.36 (1.12–1.60). However, the number of GI infections registered among the participant puppies during the whole assay varied depending on whether or not they had received supplementation and the type of probiotic received, and the breed (multifactorial ANOVA; Table 4). On the other hand, no differences were found between male and female puppies within each breed; therefore, the number of GI infections was jointly analyzed for both sexes.

**Table 4:** Effects and interactions of probiotic supplementation (control/*L. plantarum* MP02 group/*L. rhamnosus* MP01 group), breed (German shepherd/Yorkshire) and sex (female/male) on the number of gastrointestinal infections of the participant puppies during the first 4 weeks (weeks 5–8 of age) and the last 4 weeks (weeks 9–12 of age) of the assay as determined by two-way ANOVA tests.

	Weeks 5–8 of age		Weeks 9–12 of age	
Effect	*F*-value	*p*-value	*F*-value	*p*-value
Probiotic type	0.13	0.876	29.57	0.000
Breed	2.68	0.107	12.35	0.001
Sex	0.03	0.856	0.41	0.525
Probiotic type × Breed	0.40	0.674	0.33	0.719
Probiotic type × Sex	0.13	0.876	0.18	0.837
Breed × Sex	0.30	0.587	3.67	0.060
Probiotic type × Breed × Sex	0.00	1.000	0.23	0.796

The probiotic supplementation did not change the number of mean GI infections recorded between weeks 5 and 8 of the assay (Table 5). The mean (95% CI) number of GI infections on German shepherd puppies was 0.83 (0.55–1.12) for the C group and 0.92 (0.63-1.20) for both probiotic groups (LP and LR) in the first 4 weeks of the assay (one-way ANOVA: *F* = 0.06, *p*-value = 0.943). In contrast, there was a statistically significant decrease in the onset of new GI infections between weeks 9 and 12 of the assay (Table 5). The mean (95% CI) number of GI infections in the control group of German shepherd puppies increased to 2.50 (2.19–2.81) in the last 4 weeks of the assay, while in the probiotic groups the occurrence was lower, i.e., 1.42 (1.10–1.73) and 1.08 (0.77–1.40) new episodes per individual for the LP and LR groups, respectively (one-way ANOVA: *F* = 11.54, *p*-value = 0.001) (Table 5). Similar results were noted for Yorkshire puppies (Table 5). Although the mean number of GI infections in the probiotic LR groups was lower than in the probiotic LP groups of both breeds, these differences did not reach statistical significance (Table 5).

**Table 5:** Gastrointestinal infection episodes in German shepherd and Yorkshire puppies supplemented or not with the probiotics *L plantarum* MP02 or *L. rhamnosus* MP01 during the first 4 weeks (weeks 5–8 of age) and the last 4 weeks (weeks 9–12 of age) of the assay in a canine experimental model.

			Weeks 5–8 of age		Weeks 9–12 of age		Total (weeks 5–12)	
Breed	Group^a^	n	Mean (95% CI)	*p*-value^b^	Mean (95% CI)	*p*-value^b^	Mean (95% CI)	*p*-value^b^
German shepherd	C	12	0.83 (0.55–1.12)	0.943	2.50 (2.19–2.81)a	<0.001	3.33 (2.81–3.85)a	0.035
	LP	12	0.92 (0.63–1.20)		1.42 (1.10–1.73)b		2.33 (1.82–2.85)ab	
	LR	12	0.92 (0.63–1.20)		1.08 (0.77–1.40)b		2.00 (1.48–2.52)b	
								
Yorkshire	C	12	0.75 (0.52–0.98)	0.530	2.08 (1.79–2.38)a	0.000	2.83 (2.37–3.30)a	< 0.001
	LP	12	0.67 (0.44–0.89)		0.67 (0.37–0.96)b		1.33 (0.87–1.80)b	
	LR	12	0.50 (0.27–0.73)		0.42 (0.12–0.71)b		0.92 (0.45–1.38)b	

*CI, confidence interval. ^a^C, Control group; LP, group supplemented with L. plantarum MP02; LR, group supplemented with L. rhamnosus MP01. ^b^One-way ANOVA tests to compare the mean weight of the puppies at each time interval followed by Tukey’s post hoc tests; different letters besides mean values represent significant differences between groups (p < 0.05).*

**FIGURE 3:**
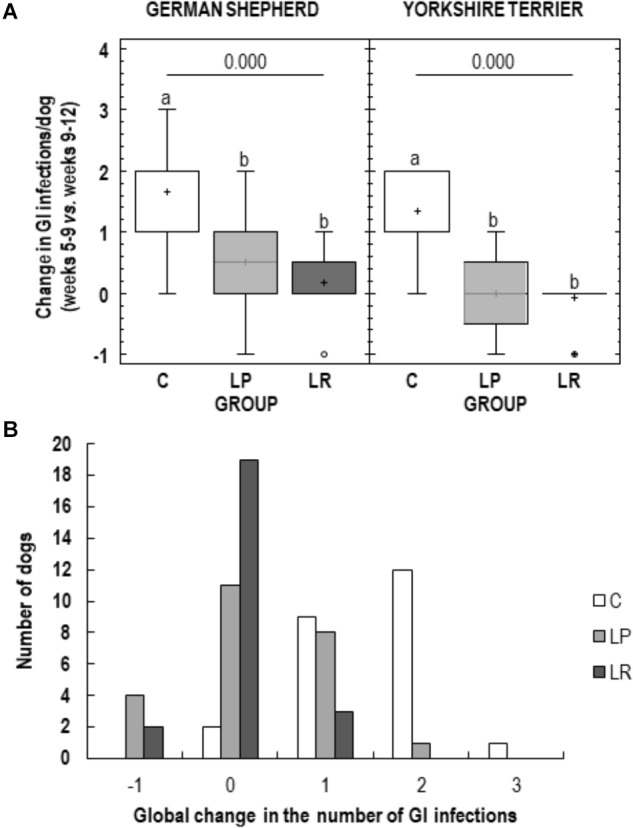
Clinical evolution of German shepherd and Yorkshire puppies during the assay. **(A)** Change in the number of gastrointestinal infection (GI) episodes registered for each puppy during the last four weeks of the assay (weeks 9–12 of age) with respect to the number of GI episodes recorded during the first four weeks of the assay (weeks 5–8 of age) in the control group (C, white), in the *L. plantarum* MP02 group (LP, light grey) and in the *L. rhamnosus* MP01 group (LR, deep gray). **(B)** Global change in the number of GI episodes in the three study groups (C, control group; LP, group supplemented with *L. plantarum* MP02; LR, group supplemented with *L. rhamnosus* MP01).

The clinical evolution of the puppies was evaluated comparing the number of GI infection episodes experienced during the last 4 weeks of the assay (weeks 9–12) with those registered during the first 4 weeks (weeks 5–8), both individually and for the whole group of animals included in this study (Figure 3). High interindividual variability in the clinical evolution of the participant puppies in both control and LP groups was observed, in contrast to LR (Figure 3). Although the mean (95% CI) increase in GI infections was 0.17 (−0.20-0.53) episodes per animal in group LR and 0.5 (−0.01 – 1.01) in the LP group of German shepherd puppies, the difference was not statistically significant (paired *t*-test; *p* = 0.253). Similarly, in Yorkshire puppies, the mean (95% CI) change in GI infections was – 0.08 (−0.27 – 0.10) in group LR and of 0.00 (−0.47 – 0.47) in group LP (paired *t*-test; *p* = 0.719) (Figure 3).

Most of the participant puppies (92%; 22 out of 24) in the C group experienced one (*n* = 9) or two (*n* = 12) more episodes of GI infection during the last 4 weeks of the assay compared to those registered during the first 4 weeks of the assay; and one Yorkshire puppy suffered 3 more episodes. The number of participant puppies having new GI infections during the second period of the assay decreased to 38% (9 out of 24) and 13% (3 out of 24) in the LP and LR groups, respectively. It is worth stressing that 79% of the puppies (19 out of 24) in the LR group did not experience new episodes of GI infection in the second period of the assay. On the other hand, a reduction in the number of GI infections in this last period of the assay was only observed in 6 participants belonging to groups LP and LR. The differences observed between the three experimental groups regarding the change in the number of GI infection episodes registered was statistically significantly different (Fisher’s exact test; *p* < 0.001).

**FIGURE 4:**
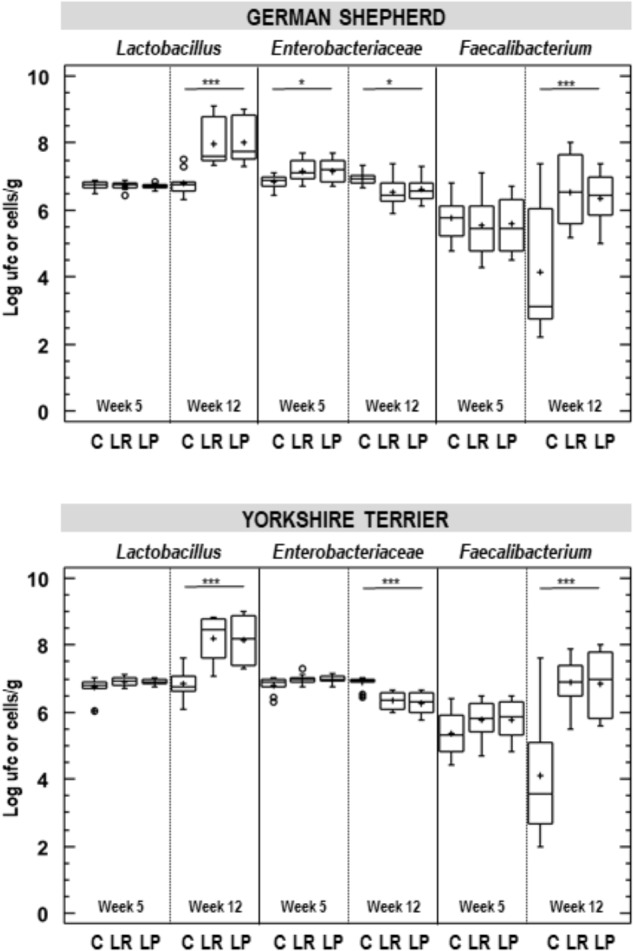
Fecal bacterial counts in German shepherd and Yorkshire puppies at the beginning (week 5) and at the end (week 12) of the assay in the control (C), *L. plantarum* MP02 (LP) and *L. rhamnosus* MP01 (LR) groups. *Lactobacillus* and *Enterobacteraceae* counts were determined by conventional plating and *Faecalibacterium* content was estimated by quantitative real time-PCR. ^∗^*p* < 0.050; ^∗∗∗^*p* < 0.001 (one-way ANOVA).

The effect of probiotic supplementation on selected intestinal bacterial groups (*Lactobacillus*, *Enterobacteriaceae* and *Faecalibacterium*) was also analyzed (Supplementary Table S2). At the beginning of the assay, at week 5 of age, median (IQR) bacterial counts in feces were 6.79 (6.73–6.83) and 7.00 (6.93–7.06) log cfu/g for *Lactobacillus* and *Enterobacteriaceae*, while the mean (95% CI) bacterial content for *Faecalibacterium* was 5.63 (5.47–5.80) log cells/g, although small differences were found for *Lactobacillus* and *Enterobacteriaceae* (about 0.12 log cfu/g) depending on the breed (Figure 4). A small difference in *Enterobacteriaceae* counts was also observed in German shepherd puppies depending on the group: those in the control group had lower *Enterobacteriaceae* counts (about 0.33 log cfu/g) than those participants in both probiotic groups (one-way ANOVA; *p* = 0.018; Table 6 and Figure 4).

**Table 6:** Fecal bacteria (log [cfu or cells]/g) in German shepherd and Yorkshire puppies supplemented or not with the probiotics *L plantarum* MP02 or *L. rhamnosus* MP01 at the beginning (5 weeks of age) and at the end (12 weeks of age) of the assay in a canine experimental model and the change at individual level during the assay.

				5 weeks of age		12 weeks age		Individual change during the assay	
									
Breed	Bacterial group	Group^a^	*n*	Mean (95% CI)^b^	*p*-value^c^	Mean (95% CI)	*p*-value	Mean (95% CI)	*p*-value
German shepherd	*Lactobacillus*	C	12	6.75 (6.70–6.80)	0.857	6.78 (6.54–7.03)a	0.000	0.04 (0.17 – −0.20)a	0.000
		LP	12	6.72 (6.68–6.77)		8.02 (7.78–8.27)b		1.30 (0.17 – 1.06)b	
		LR	12	6.74 (6.69–6.78)		8.00 (7.75–8.26)b		1.26 (0.17 – 1.02)b	
	*Enterobacteriaceae*	C	12	6.85 (6.72–6.98)a	0.018	6.93 (6.80–7.07)a	0.012	0.08 (−0.02 – 0.19)a	0.000
		LP	12	7.18 (7.05–7.30)b		6.61 (6.48–6.75)b		−0.56 (−0.67 – −0.46)b	
		LR	12	7.18 (7.05–7.31)b		6.52 (6.39–6.66)b		−0.66 (−0.76 – −0.55)	
	*Faecalibacterium*	C	12	5.75 (5.43–6.07)	0.792	4.14 (3.58–4.70)a	<0.001	−1.60 (−2.17 – −1.05)a	0.000
		LP	12	5.58 (5.25–5.90)		6.38 (5.81–6.94)b		0.80 (0.24 – 1.36)b	
		LR	12	5.55 (5.23–5.87)		6.56 (6.00–7.12)b		1.01 (0.45 – 1.57)b	
Yorkshire	*Lactobacillus*	C	12	6.76 (6.69–6.83)	0.085	6.84 (6.58–7.10)a	0.000	0.08 (−0.19 – 0.36)a	<0.001
		LP	12	6.90 (6.83–6.97)		8.17 (7.90–8.43)b		1.28 (1.01 – 1.55)b	
		LR	12	6.90 (6.83–6.98)		8.18 (7.92–8.45)b		1.27 (0.99 – 1.54)b	
	*Enterobacteriaceae*	C	12	6.87 (6.82–6.93)	0.080	6.87 (6.76–6.98)a	0.000	0.05 (−0.05 – 0.16)a	0.000
		LP	12	6.99 (6.92–7.06)		6.26 (6.15–6.37)b		−0.64 (−0.74 – −0.54)b	
		LR	12	6.98 (6.91–7.05)		6.33 (6.23–6.45)b		−0.73 (−0.83 – −0.63)b	
	*Faecalibacterium*	C	12	5.38 (5.12–5.63)	0.193	4.09 (3.54–4.64)a	0.000	−1.28 (−1.81 – −0.75)a	0.000
		LP	12	5.78 (5.53–6.04)		6.83 (6.28–7.39)b		1.13 (0.60 – 1.65)b	
		LR	12	5.77 (5.51–6.02)		6.89 (6.34–7.44)b		1.05 (0.52 – 1.58)b	

*CI, confidence interval. ^a^C, Control group; LP, group supplemented with L. plantarum MP02; LR, group supplemented with L. rhamnosus MP01. ^b^Lactobacillus and Enterobacteriaceae content is expressed as log cfu/g and Faecalibacterium content as log cell/g. ^c^One-way ANOVA; different letters indicate which groups are statistically significant different.*

The supplementation with any of the probiotics led to a substantial change in the fecal content of these bacterial groups. First, *Lactobacillus* counts in feces increased more than one log unit in both breeds when the probiotic was administered (Figure 4). For German shepherd puppies, the mean raise in fecal *Lactobacillus* counts experienced by each participant varied between 1.26 and 1.30 log cfu/g for those supplemented with *L. rhamnosus* MP01 and *L. plantarum* MP02, respectively, while no change was observed for puppies in C group; the differences between the control and the probiotic groups was statistically significant (one-way ANOVA, *p* = 0.000; Table 6). Regarding bacterial counts for *Enterobacteriaceae*, there was no change in the control group during the assay, but lower counts were found in both probiotic groups (0.56 and 0.66 log cfu/g for group LP and LR, respectively); the individual change registered for *Enterobacteriaceae* in the control and the probiotic groups was also statistically significant (one-way ANOVA, *p* = 0.000; Table 6). The variability in fecal *Faecalibacterium* content was higher than for *Lactobacillus* and *Enterobacteriaceae* in all groups (Figure 4). There was a decrease (1.60 log cells/g) in fecal *Faecalibacterium* counts in the control group at the end of the assay, while the opposite was found for both probiotic groups (an increase of 0.80 and 1.01 log cell/g for groups LP and LR, respectively) (Table 6). Similar results were registered for the corresponding groups of Yorkshire puppies (Figure 4 and Table 6).

**FIGURE 5:**
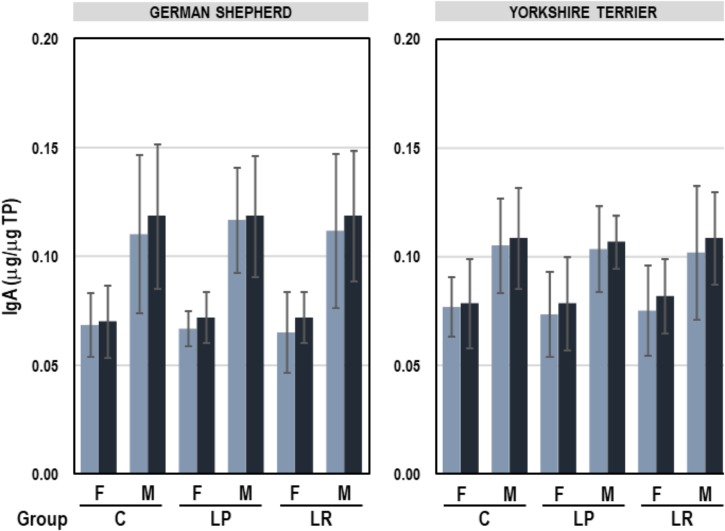
IgA content (μg/μg TP) in feces of German shepherd and Yorkshire female (F) and male (M) puppies at the beginning (week 5, light blue) and at the end (week 12; dark blue) of the assay in the control (C), *L. plantarum* MP02 (LP) and *L. rhamnosus* MP01 (LR) groups. Paired *t*-tests were performed to analyze if the individual change in the concentration of IgA at the end of the assay was statistically significant in each group of dogs.

The mean (95% CI) concentration of IgA in feces was 0.09 (0.09–0.10) μg IgA/μg TP, ranging from 0.04 to 0.18 μg IgA/μg TP. A multifactorial ANOVA analysis was performed to assess the influence of breed, sex and group (C, LP, and LR) on the fecal IgA content. The only variable that had a significant effect on fecal IgA concentration was sex (*F* = 45.39, *p* = 0.000; multifactorial ANOVA test). IgA levels were higher in feces of male than in those of female puppies (average 0.11 vs. 0.07 μg IgA/μg TP for male and female, respectively), but the administration of probiotics for 8 weeks did not have any effect on this parameter (Figure 5). In addition, there was no correlation between fecal IgA content and the concentration of *Lactobacillus*, *Enterobacteriaceae*, or *Faecalibacterium* (Table 7).

**FIGURE 6:**
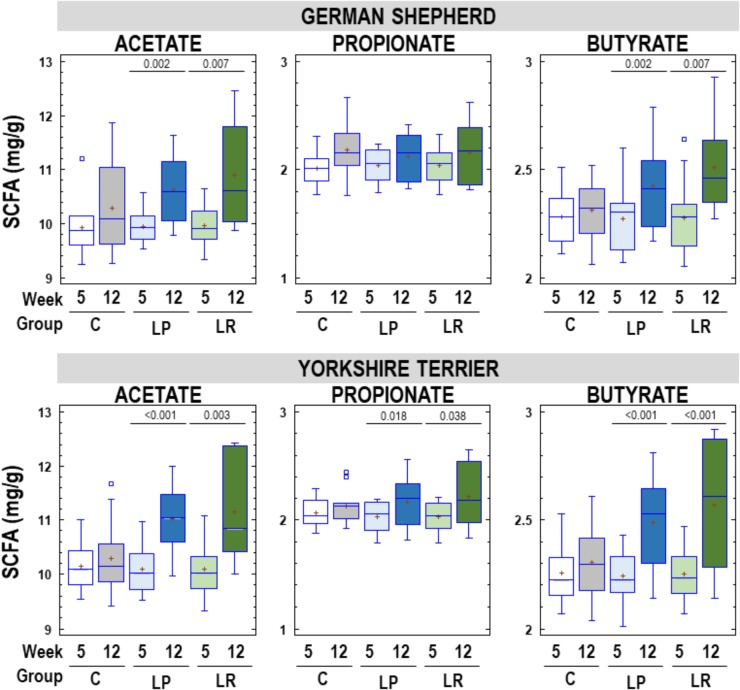
Fecal SCFAs (acetate, propionate, and butyrate) content (mg/g) in feces of German shepherd and Yorkshire puppies at the beginning (week 5) and at the end (week 12) of the assay in the control (C), *L. plantarum* MP02 (LP) and *L. rhamnosus* MP01 (LR) groups. Paired *t*-tests were performed to analyze if the individual change in the concentration of individual SCFAs at the end of the assay was statistically significant for each group of dogs.

The most abundant SCFA in fecal samples was acetate, that ranged from 9.24 to 12.46 mg/g, followed by butyrate (range = 2.01-2.93 mg/g) and propionate (range = 1.77–2.67 mg/g). Acetate, propionate and butyrate concentrations in feces of German shepherd and Yorkshire puppies in C, LP, and LR groups at the beginning and at the end of the assay are shown in Figure 6. Globally there was an increase in fecal SCFA content in all participant puppies at the end of the assay, but the mean rise in the concentration of fecal SCFA was statistically significant only in the probiotic groups for all SCFAs, except for propionate in German shepherd puppies. The mean increase in fecal acetate concentration was 0.36, 0.68 and 0.94 mg/g of feces (about 4, 7, and 9%) for C, LP, and LR groups, respectively, in German shepherd puppies. Similar percentages of increase were found for butyrate (1.5, 7, and 10% for C, LP, and LR groups, respectively) in this breed. The same trend was observed in Yorkshire puppies regarding fecal content of SCFAs, although the increase was slightly higher than in German shepherd puppies. In the control group of Yorkshire puppies, fecal acetate, propionate and butyrate increased by 0.15, 0.06, and 0.05 mg/g of feces, respectively, which represented approximately 1.5, 3, and 2% of increase. In contrast, in the LP group of Yorkshire puppies, the fecal concentration of SCFAs raised by 9, 7, and 11% for acetate, propionate and butyrate; and in the LR group of this breed, the increase reached 10.5, 9, and 14%, respectively.

There was a moderate positive correlation between butyrate content and *Lactobacillus* counts (ρ = 0.524; *p* = 0.000) and *Faecalibacterium* cells (ρ = 0.544; *p* = 0.000) in feces as well as between acetate content and *Lactobacillus* counts (ρ = 0.441; *p* = 0.000). In contrast, *Enterobacteriaceae* counts showed a moderate negative relationship with both acetate (ρ = −0.454; *p* = 0.000) and butyrate (ρ = −0.499; *p* = 0.000) concentration in feces. There was no correlation between the content in feces of any SCFA and IgA (Table 7).

**Table 7:** Spearman correlations between fecal bacterial counts of *Lactobacillus* and *Enterobacteriaceae*, and *Faecalibacterium* content and the concentration in feces of IgA and short chain fatty acids (acetate, propionate, and butyrate).

	*Enterobacteriaceae*	*Faecalibacterium*	IgA	Acetate	Propionate	Butyrate
*Lactobacillus*	−0.485^a^	0.608	0.058	0.441	0.290	0.524
	*0.000*	*0.000*	*0.490*	*0.000*	*0.001*	*0.000*
*Enterobacteriaceae*		−0.486	0.122	−0.454	−0.132	−0.499
		*0.000*	*0.145*	*0.000*	*0.114*	*0.000*
*Faecalibacterium*			−0.094	0.282	0.175	0.544
			*0.262*	*0.001*	*0.036*	*0.000*
IgA				0.063	0.210	−0.102
				*0.451*	*0.012*	*0.223*
Acetate					0.432	0.293
					*0.000*	*0.001*
Propionate						0.401
						*0.000*

*The p-value showing the statistical significance of the estimated correlation is shown in italics below the Spearman’s correlation coefficient. ^a^Spearman’s rank correlation coefficient (ρ).*

## Discussion

In this study, the probiotic potential of two lactobacilli strains isolated from canine milk was investigated, both *in vitro* and *in vivo*, including an assay in a canine experimental model in order to evaluate their potential to prevent gastroenteritis. Our results confirm that lactobacilli are naturally present in canine milk and, therefore, this biological fluid provides a natural and continuous source of such microorganisms for the suckling puppy. In addition to bitches, lactobacilli strains have been also isolated from milk of other mammalian species, including sows and women (Martín et al., 2003, 2009, 2010).

The two lactobacilli strains isolated in this work belong to the species *L. rhamnosus* and *L. plantarum* that have the qualified presumption of safety (QPS) status of the EFSA (European Food Safety Authority [EFSA], 2017) and are included in a variety of commercial probiotic preparations; in fact, some strains have already been tested as canine probiotics (Scott Weese and Anderson, 2002; Manninen et al., 2006; Biagi et al., 2007). Consequently, and as it has been already observed in other mammalian species, lactobacilli isolated from milk of healthy bitches have potential interest as probiotics displaying a protective effect in both mothers and/or puppies against infectious diseases. So far, most of the commercial probiotic strains for dogs do not have a canine origin. Findings of a previous study showed that a human isolate of *Lactobacillus* (*L. rhamnosus* GG) survived GI transit in dogs but it was not efficient regarding fecal colonization as it has been reported in humans (Scott Weese and Anderson, 2002).

Since lactobacilli strains isolated from canine milk have an attractive origin for canine applications, the two lactobacilli strains were screened for the presence of a variety of prerequisite properties for probiotic bacteria, such as antimicrobial activity, adherence to epithelial cells and mucin, survival when exposed to adverse conditions that can be found in the GI tract and sensitivity to antibiotics. Both strains displayed high antimicrobial activities against canine pathogens originally isolated from gastroenteritis cases and high survival rates after exposure to GI-like conditions. In addition, these strains were strongly adhesive to both Caco-2 and HT-29 cells, did not degrade mucin, and the MICs of several antibiotics were within the values recently recommended by EFSA (European Food Safety Authority [EFSA], 2018). Such results are similar to those obtained with lactobacilli strains isolated from human and canine milk (Martín et al., 2005, 2010).

Lactation is a critical period in canine breeding and, consequently, early weaning is an important cause of puppy mortality and morbidity. Canine milk offers a rich source of all the nutrients and energy required for the rapidly growing puppy and, at the same time, exerts protection against infectious diseases when the extra-uterine life starts. This protective effect is related to the concerted action of a variety of protective factors present in colostrum and mature milk including immunoglobulins, immunocompetent cells, fatty acids with antimicrobial activity, polyamines, fucosylated oligosaccharides, lysozyme, lactoferrin and commensal bacteria. At the present time, microbiological characterization of canine milk has been performed only in exceptional circumstances in order to identify potential pathogenic bacteria in clinical perinatal infections, such as lactational mastitis in bitches and septicaemia in neonatal puppies (Jung et al., 2002; Schäfer-Somi et al., 2003; Ververidis et al., 2007). In contrast, well documented studies show that human milk is an excellent and continuous source of probiotic lactic acid bacteria to the infant gut, which may play an important role in reducing the incidence and severity of infections in the breastfed infant (Fernández et al., 2013).

Interestingly, the ability of some lactic acid bacteria strains isolated from human milk of healthy women to inhibit a wide spectrum of pathogenic bacteria was reported in a previous study (Martín et al., 2005). The strains isolated and characterized in this study seem to have similar antimicrobial properties against different bacterial species involved in canine gastroenteritis; however, the antiinfectious ability of potentially probiotic strains must be tested in *in vivo* experimental models or in clinical trials. As a consequence, both strains were assayed in a canine experimental model to elucidate their probiotic potential in the prevention of gastroenteritis among male and female puppies belonging to two very different breeds (German shepherd and Yorkshire) living and kennels and, thus, being particularly prone to such kind of infections. The rates of reductions in GI infections observed in both probiotic groups and in both canine breeds are comparable to those achieved in human studies that reported a successful prevention of infant GI infections or diarrhea episodes using a probiotic infant formula (Weizman et al., 2005; Picaud et al., 2010; Maldonado et al., 2012). Interestingly, the later study (Maldonado et al., 2012) was carried with a *Lactobacillus* strain previously isolated from human milk.

Fecal samples were analyzed to investigate some of the potential mechanisms responsible for the reduction in such infections. Similarly to other studies, no significant differences in IgA concentrations were observed among the control and the probiotic groups (Vilson et al., 2018). However, the intake of both probiotic strains resulted in a significant increase in the *Lactobacillus* and *Faecalibacterium* counts in feces and, also, in a significant increase in the fecal concentrations of SCFAs. These changes in the gut microbiota could, at least in part, explain the reductions in the number of gastroenteritis episodes observed in the probiotic groups. The increase in butyrate-producing bacteria, such as *Faecalibacterium* (Guard et al., 2015) may be the result of the metabolic activity of lactobacilli and, particularly to their ability to produce lactic acid, which may favor the growth and production of butyrate by gut-associated strict anaerobes. The genus *Faecalibacterium* seems to be an excellent biomarker of good canine GI health since its concentration is notably reduced in dogs with acute diarrhea, colon tumors, and chronic inflammatory enteropathies (CE) compared to healthy dogs (Suchodolski et al., 2012; Guard et al., 2015; Suchodolski, 2016; AlShawaqfeh et al., 2017; Gavazza et al., 2018; Herstad et al., 2018).

It has been previously reported that the population of other butyrate-producers (such as bifidobacteria) significantly increases after supplementation with lactobacilli (Gueimonde et al., 2006; Sierra et al., 2010; Maldonado et al., 2012). Butyrate reinforces the gut barrier and protects against gastroenteritis by inducing apoptosis in cells with damaged DNA, and also by increasing the expression of the genes encoding tight junction proteins (Wong et al., 2006; Antharam et al., 2013). Propionic acid is another SCFA with potential protective effects against carcinogenesis and colorectal cancer in humans (Hinnebusch et al., 2002) and statistically significant changes in propionate concentrations were also observed after probiotic supplementation in this study. The level of lactobacilli and that of butyrate-producing bacteria is substantially reduced in infants with colic compared with healthy infants (de Weerth et al., 2013). Notably lactobacilli reduction was found to be limited to species considered to be mucosal lactobacilli, including *L. plantarum*, and it has been found that exponentially-growing *L. plantarum* cells induce expression of antiinflammatory genes in the human upper intestinal tract (van Baarlen et al., 2009). In contrast, no effect upon the diversity or composition of the gut canine microbiota was observed after pre- and postnatal exposure of bitches and puppies to *Lactobacillus johnsonii* NCC533 (La1), a strain of human origin used without a previous assessment of target-specific properties for canine populations (Vilson et al., 2018).

## Conclusion

Considering the significant decrease in the number of infections, the administration of the probiotic strains isolated and characterized in this study may be useful for the prevention of GI infections in dogs. The decrease in *Faecalibacterium* levels and the fecal dysbiosis present in acute and chronic intestinal diseases of dogs have been repeatedly associated with altered systemic metabolic states, mainly alterations in SCFAs concentrations (Guard et al., 2015). As previously stated, this fact highlights the importance of dysbiosis in the pathophysiology of GI diseases, and may also lead to new diagnostic and therapeutic approaches (Suchodolski, 2016), including the use of target-specific and well characterized probiotics.

Work is in progress to initiate well-designed clinical trials in order to confirm the efficacy of the strains in the prevention of canine GI disorders.

## Ethics Statement

All animals were treated in strict accordance with the guidelines of the European Directive 2010/63/UE on the protection of animals used for scientific purposes. The study was approved by Ethical Committee on Animal Experimentation of Universidad Complutense de Madrid (Spain), under protocol 29/17.

## Author Contributions

JR and LF conceived and designed the experiments. RM, MP, LF, and RA performed the experiments. JR and LF analyzed the data and wrote the manuscript. All the authors approved the submitted version of the manuscript.

## Conflict of Interest Statement

The authors declare that the research was conducted in the absence of any commercial or financial relationships that could be construed as a potential conflict of interest.

## Footnotes

1 http://www.physics.csbsju.edu/stats/
