# Supplementary material for: Characterization of Lactobacillus rhamnosus MP01 and Lactobacillus plantarum MP02 and Assessment of Their Potential for the Prevention of Gastrointestinal Infections in an Experimental Canine Model
Source: Front Microbiol. 2019 May 24;10:1117. doi: 10.3389/fmicb.2019.01117 (PMC6543525; doi:10.3389/fmicb.2019.01117)
Supplement: Supplementary file 1 [file Table_1.DOCX]

**Table S1.** Effects and interactions of probiotic supplementation (control group/*L. plantarum* MP02 group/*L. rhamnosus* MP01 group) and sex on the weight gain (kg) of the participant puppies during the trial as determined by two-way ANOVA tests.

| **Breed** | **Effect** | ***F-value*** | ***P-value*** |
| --- | --- | --- | --- |
| German shepherd | Probiotic type | 5.17 | 0.012 |
|  | Sex | 26.05 | 0.000 |
|  | Probiotic type × Sex | 0.16 | 0.856 |
| Yorkshire | Probiotic type | 1.61 | 0.216 |
|  | Sex | 55.06 | 0.000 |
|  | Probiotic type × Sex | 0.03 | 0.972 |
