# Supplementary material for: Characterization of Lactobacillus rhamnosus MP01 and Lactobacillus plantarum MP02 and Assessment of Their Potential for the Prevention of Gastrointestinal Infections in an Experimental Canine Model
Source: Front Microbiol. 2019 May 24;10:1117. doi: 10.3389/fmicb.2019.01117 (PMC6543525; doi:10.3389/fmicb.2019.01117)
Supplement: Supplementary file 2 [file Table_2.DOCX]

**Table S2.** Effects and interactions of probiotic supplementation (control/*L. plantarum* MP02 group/*L. rhamnosus* MP01 group) and breed (German shepherd/Yorkshire) on the fecal counts of selected bacterial groups at the beginning (5 weeks of age) and at the end (12 weeks of age) of the trial as determined by two-way ANOVA tests.

|  | **5 weeks of age** | | **12 weeks of age** | |
| --- | --- | --- | --- | --- |
| **Effect** | ***F*-value** | ***p*-value** | ***F*-value** | ***p*-value** |
| ***Lactobacillus*** |  |  |  |  |
| Probiotic type | 1.40 | 0.254 | 34.88 | 0.000 |
| Breed | 11.64 | 0.001 | 0.78 | 0.380 |
| Probiotic type × Breed | 2.43 | 0.096 | 0.06 | 0.940 |
| ***Enterobacteriaceae*** |  |  |  |  |
| Probiotic type | 6.87 | 0.002 | 19.87 | 0.000 |
| Breed | 4.70 | 0.034 | 7.84 | 0.007 |
| Probiotic type × Breed | 1.64 | 0.203 | 0.97 | 0.384 |
| ***Faecalibacterium*** |  |  |  |  |
| Probiotic type | 0.18 | 0.832 | 28.86 | 0.000 |
| Breed | 0.01 | 0.920 | 0.61 | 0.437 |
| Probiotic type × Breed | 1.42 | 0.250 | 0.27 | 0.765 |
